# Parental Identity and Lived Experiences when Parenting a LGBTQIA+2 Child: A Critical Narrative Synthesis of Current Evidence

**DOI:** 10.1080/17482631.2024.2378511

**Published:** 2024-07-14

**Authors:** Cherryl S. Kolhe, Shirin Shikalgar, Deborah Biggerstaff

**Affiliations:** a Symbiosis Centre for International Education, Symbiosis International (Deemed) University, Pune, India; b Symbiosis Community Outreach Program Extension (SCOPE), Symbiosis International (Deemed) University, Pune, India; c Mental Health and Wellbeing, Directorate Warwick Applied Health, Warwick Medical School, University of Warwick, Coventry, UK

**Keywords:** LGBTQIA+2, cisgender, heterosexual parenting experiences, parent identity change, lived LGBTQIA+2 parenting experiences, identity process theory, qualitative narrative synthesis

## Abstract

**Purpose:**

Most LGBTQIA + 2 studies focus on the core sexually and gender diverse population without exploring the peripheral familial perspectives. Current research needs to explore parental experiences of parenting a LGBTQIA+2 child, since parents undergo an identity change after their child’s disclosure. This parental identity change may affect parental well-being and add to the existing stress of parenting a LGBTQIA + 2 child.

**Methods:**

This paper uses the identity process theory (IPT) to review 18 studies on parental narratives to highlight the change in parental identity. Databases were searched for first-hand parenting experiences and shortlisted articles were qualitatively synthesized.

**Findings:**

We identified six main themes: I) Parental identity change is triggered by a child’s disclosure. II) Parental identity change drives parental emotions that evolve from initial anger, shock, fear, concern, grief, etc. to eventual acceptance of their child. III) Parental identity and emotions change, as for any life change process, across assimilation, accommodation, adjustment, and evaluation phases of the IPT. IV) Parental identity change is motivated by continuity, coherence, self-efficacy, belongingness, distinctiveness, meaning and self-esteem principles of the IPT. V) Parental identity influences parental micro-individual, meso-interactional and macro-societal interactions of the IPT framework. VI) Parental mental well-being may be affected across the assimilation, accommodation, adjustment phases of the IPT before eventual acceptance of the child in the evaluation phase.

**Conclusions:**

Parental lived experiences require a stronger consideration today within the wider, non-white, contexts. The effect of identity change on parental mental well-being and its intergenerational effect needs to be explored within the context of the IPT. Parental narratives will contribute towards creating appropriate counselling toolkits and interventions for health care providers and parents of LGBTQIA + 2 children.

## GRAPHICAL ABSTRACT

**Figure:**
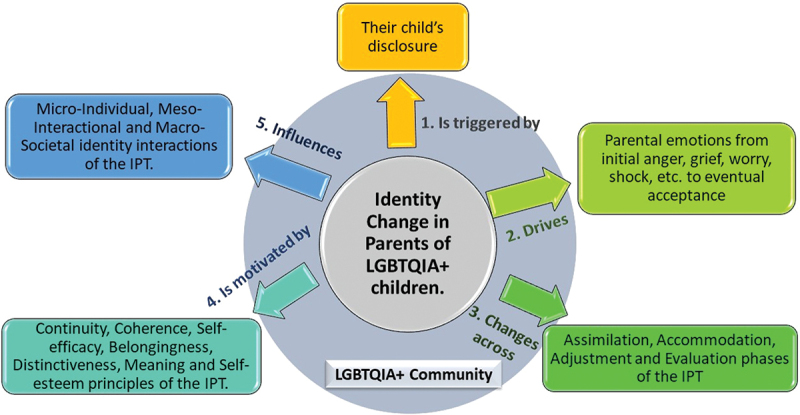

## 1. Introduction

The gender and sexual minority representation in global statistics today substantiates the acknowledgement of the LGTBQIA + 2 cohort in society (Ayhan et al., 2020; McGeough & Sterzing, 2018). According to the 2021 LGBT Pride Global Survey Report which collates data from 27 countries, an average 80% of the global population surveyed identifies as heterosexual. Among the remainder 20%, 3% identify as “homosexuals”, which includes gays and lesbians, 4% identify as “bisexuals”, 1% each as “pansexual” or “omnisexual”, “asexual” or others. Eleven percent either do not know what to say or do not divulge information about their sexual orientation (LGBT Pride 2021 Global Survey Report). In terms of gender identity, an average 1% of the global population describe their gender identity as “fluid”. This group identifies as “transgender,” “non-binary/non-conforming/gender-fluid” or “in another way” other than as “male” or “female” (LGBT Pride 2021Global Survey Report). These changes in gender and sexual minority representation indicate how today’s LGBTQIA + 2 community is gaining momentum as a mainstream research entity across both health and society.

Over recent decades, health and social research has become increasingly concerned about highlighting the social, emotional, physical, financial and psychological needs of the LGBTQIA + 2 community (Flores et al., 2021; Grafsky, 2014; Le et al., 2016; McGeough & Sterzing, 2018; Mehus et al., 2017; Murphy, 2018; Sequeira et al., 2020; Watson et al., 2019). More recently, researchers have gravitated towards studying the family interactions of the LGBTQIA + 2 community focusing closely on the needs of families, especially their perspectives on reactions to disclosure and post-disclosure events occurring in the home (Abreu et al., 2022; Gyamerah et al., 2019; Martin et al., 2010; Mills-Koonce et al., 2018; Newhook et al., 2018; Rosati et al., 2020; Thornburgh et al., 2020). Parental studies examining family dynamics, especially those considering parental experiences of parenting a young person who identifies as LGBTQIA + 2 have recently begun to receive greater traction in the research field. This is attributed to the complex nature of parental challenges that may come with the child’s realization and disclosure of their sexual and/or gender identity followed by the transitions. For the majority of families today, many witness the disclosure and post disclosure events more closely and may face the major consequential brunt of these transitionary stages (Wagner & Armstrong, 2020). Social norms may also add to parents’ existing parenting challenges as they navigate through what is to them an unchartered territory. Many parents assume that their children will conform to the current gender and sexuality norms of their society (Katz-Wise et al., 2016; Stotzer, 2011). Such disclosures may therefore catch parents “off-guard”, thus leading to any such parental “unpreparedness” making the disclosure and its following events stressful. However, parental assumptions about their child’s heteronormativity and being cisgender is perceived by many to be an uncritically accepted fact (Carnelley et al., 2011; Littman & Romer, 2018; Spivey et al., 2018). Any such child’s disclosure may also alter the parent’s identity as they acknowledge and adapt themselves to the demands of a new parenting arena (G. Breakwell, 2015; Phillips & Ancis, 2008). While dealing with personal challenges such as adjusting to the change, parents are also expected to deal with social challenges and expectations such as informing extended family and negotiating professional and social circles. This puts parents under tremendous stress and may affect their mental well-being (Bratt et al., 2019).

Despite the pressure associated with parenting a LGBTQIA + 2 child, first-hand parental perspectives are less well documented in literature (Grafsky, 2014; Gray et al., 2016; Jaspal, 2020; Kidd et al., 2021; Larson, 2021; Littman & Romer, 2018; Saltzburg, 2004; Van Bergen et al., 2021). Particularly, in more conventional cultures, documenting first-hand experiences of parenting a sexual and gender minority child is essential to understanding the parenting phenomenon with respect to parental identity. This is because identity is a social construct grounded in a person’s upbringing, values, religion, etc. The disclosure experience for parents varies across many ethnic and particularly more “conservative” settings. Most ethnic cultures such as African, Indian, Asian, Latin-American, Hispanic, etc. may additionally be influenced by religion which can strongly impact a person’s identity, values and social standing (Gattamorta et al., 2019; Jaspal, 2020, 2017; Rothman et al., 2012; Ryan et al., 2010). In South Asian cultures such as Japanese and Chinese societies, gender is driven by social and community norms. A person’s perceived “value” is often determined by their societal and familial roles, responsibilities and contribution, which are all strongly affiliated with gender. A child’s non-conformity to their community expectations, when expressed through disclosure, can solicit unexpected and often negative parental reactions (Zhou et al., 2021). Since the definitions around gender identity and/or sexual orientation vary culturally, geographically, socially and also on religious grounds, families too may experience and react to any such disclosure with emotions that stem from their own identity, beliefs and upbringing.

This narrative review was therefore undertaken by adopting a systematic approach. It more closely explores the parental experiences, by examining the research literature documenting the lived parental experiences of parenting a LGBTQIA + 2 child. The method applies the assimilation, accommodation, adjustment and evaluation phases of the IPT to track the parental identity transition. The principles of continuity, coherence, self-efficacy, belongingness, distinctiveness, meaning and self-esteem of the IPT are employed to understand the motives behind parental identity change (Sablonnière & Usborne, 2014; Spini & Jopp, 2014). Our review also elaborates on how the change in parental identity can impact parental emotions and interactions at both individual and social levels (Chryssochoou, 2014). The narrative review aims to offer insights for strategies to prevent adverse intergenerational mental health outcomes such as suicides, substance abuse, depression, anxiety, etc. in parents and their gender and/or sexually diverse children that may arise as a result of change in parental identity (Loewenthal, 2014). We suggest that the findings of the review can contribute to facilitating practical interventions for clinicians, socio-cultural organizations and health researchers who engage with LGBTQIA + 2 children and their families (Reed et al., 2020).

## 2. Methodology

This review followed the PRISMA guidelines (Page et al., 2021) when searching and finalizing papers and adopted the PICO framework for the review process (Methley et al., 2014). This narrative approach uses the four phases, seven motivating principles and three interactions of the IPT as the guiding framework for interpreting parental identity change expressed through parental narratives.

### 2.1 Justification of the framework

**Figure 1.**
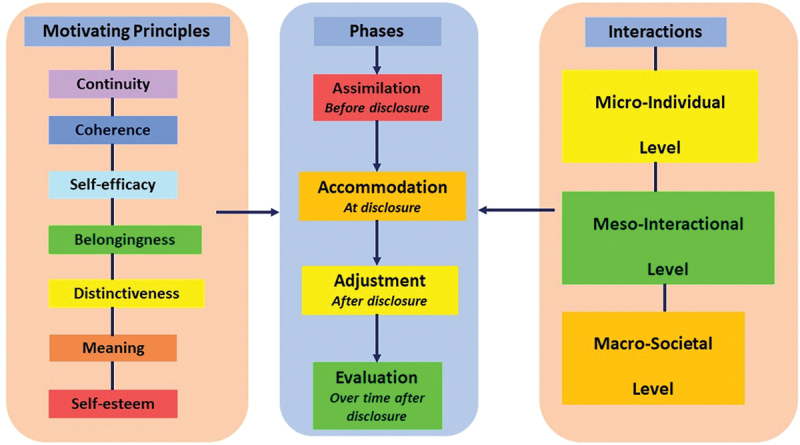
The phases, motives and interactions of the identity process theory framework.

The concept of identity has been studied in the context of the LGBTQIA + 2 population in terms of their innate identity, their identity in society, identity expression, etc. (Manning et al., 2022; Stotzer, 2011; Vázquez et al., 2023). This review shifts the identity lens more closely to focus on the perspectives of parents to explore their identity change in the context of their child’s disclosure and its implications. The disclosure event initiates a change in parental identity as parents transition into “‘becoming’ a parent of a LGBTQIA+ child” (Grafsky, 2014). The IPT offers four distinct phases that track parental identity from before, at and after disclosure in the assimilation, accommodation and adjustment phases, until parents reach eventual acceptance in the evaluation phase (Sablonnière & Usborne, 2014) (See Figure 1). The IPT also offers seven principles of continuity, coherence, self-efficacy, belongingness, distinctiveness, meaning and self-esteem that motivate the parental identity change and influence micro-individual, meso-interactional and macro-social interactions throughout the four phases of the IPT (Figure 1) (G. Breakwell, 2015; Coyle & Murtagh, 2014; Rusi et al., 2016). The simple four-phasic framework of the IPT helps track the change in parental identity through parental emotions and interactions while helping to explain the motives behind parental identity change.

### 2.2 Review Process

The review was conducted by adopting the following steps:

#### Establishing the research question

The primary research question for the review was: What research evidence is currently available regarding the lived experiences of parents in parenting their LGBTQIA + 2 children from adolescence to young adulthood, who realize and eventually disclose their gender and/or sexual non-conformity? Secondly, does this parenting experience alter the identity of parents? If so, how is any such change expressed? The research question aimed at including all the components of the PICO framework to include the P (Population), i.e., mothers and fathers of gender and/or sexual minorities, their parenting experiences as the phenomenon of Interest (I) in Co (Context) of their identity change. The definitions used for this review are detailed in Table I. Since the majority of studies we include did not take into consideration the gender and/or sexual conformity of the parents, our review relies on these studies to present narratives of heterosexual or cisgender parents.

**Table I.** Definitions used for the narrative review on the parental narratives of parenting a LGBTQIA + 2 child.

Sexuality and Gender related Definitions
**Parents**: Primary care givers, nurturing the child and involved in the child’s life before and after disclosure at the time of conducting this review. Parents may be married, divorced, biological or adopted. **Lesbian**: Women who are attracted to women (LGBT Pride 2021 Global Survey Report). **Gay**: Men who are attracted to men (Asexuality, Attraction, and Romantic Orientation., 2021). **Bisexual**: Men and women who are attracted to the same and the opposite sex (LGBT Pride n.d Global Survey Report). **Trans**: Individuals who cannot match their gender identity with their birth sex. (LGBT Pride 2021 Global Survey Report). **Queer/Questioning**: Individuals whose sexual behaviour, gender expression, or other characteristics do not conform to established social norms and who are questioning their identity (Matzner, 2022). **Intersex**: Person born with a reproductive or sexual anatomy not fitting the terms of “female” or “male.” It is an inconsistency between the external and internal genitals. For example, a baby girl with internal ovaries, and an external penis. (What Is Intersex) **Asexual**: A person experiencing no sexual feelings or desires (Asexuality, Attraction, and Romantic Orientation, 2021). **Two-Spirited**: Individuals who identify as both male and female. (IHS) **Disclosure/Coming Out**: The process wherein an individual informs his or her choice of person about their sexual and/or gender identity (Savin-Williams, 1998).
IPT Related Definitions (G. Breakwell, 2015; Chryssochoou, 2014; Rusi et al., 2016; Twigger-Ross & Uzzell, 1996)
**Identity**: An internalized and evolving narrative of self that incorporates the reconstructed past and imagined future into a coherent whole to provide the person’s life some degree of unity, purpose, and meaning. **Assimilation**: The absorption of new information into the identity structure, leading up to the event of change. (Events up to disclosure). **Accommodation**: The phenomenon of creating space for new information in the identity structure. (Disclosure). **Adjustment**: The phenomenon of making amends with the new information in the identity structure. (A few days after disclosure when parents are adjusting to the new change). **Evaluation**: A reflective phase that entails adding value and evaluating the old and new identity. (Occurs over a considerable time after disclosure, when parents reflect, evaluate and validate their parenthood). **Continuity**: To continue in the current (parental) identity. **Coherence**: To maintain compatibility between various identities. **Self-efficacy**: To remain useful in the new found (parental) identity. **Belongingness**: To claim the new (parental) identity. **Distinctiveness**: To acknowledge the uniqueness of the new (parental) identity. **Meaning**: To evaluate, find meaning, purpose, and content in the new (parental) identity. **Self-esteem**: To be proud of the new (parental) identity and enhance (parental) self-esteem. **Micro-Individual Level**: A reflective interaction with oneself. **Meso-Interactional Level**: A broader interaction with close family and social groups that are shaped by the new identity. **Macro-Societal Level**: Broad interactions with social groups, extended family and professional circles that are beyond the gender and/or sexuality diversity realm.

#### Determining inclusion and exclusion criteria of articles

**Figure 2.**
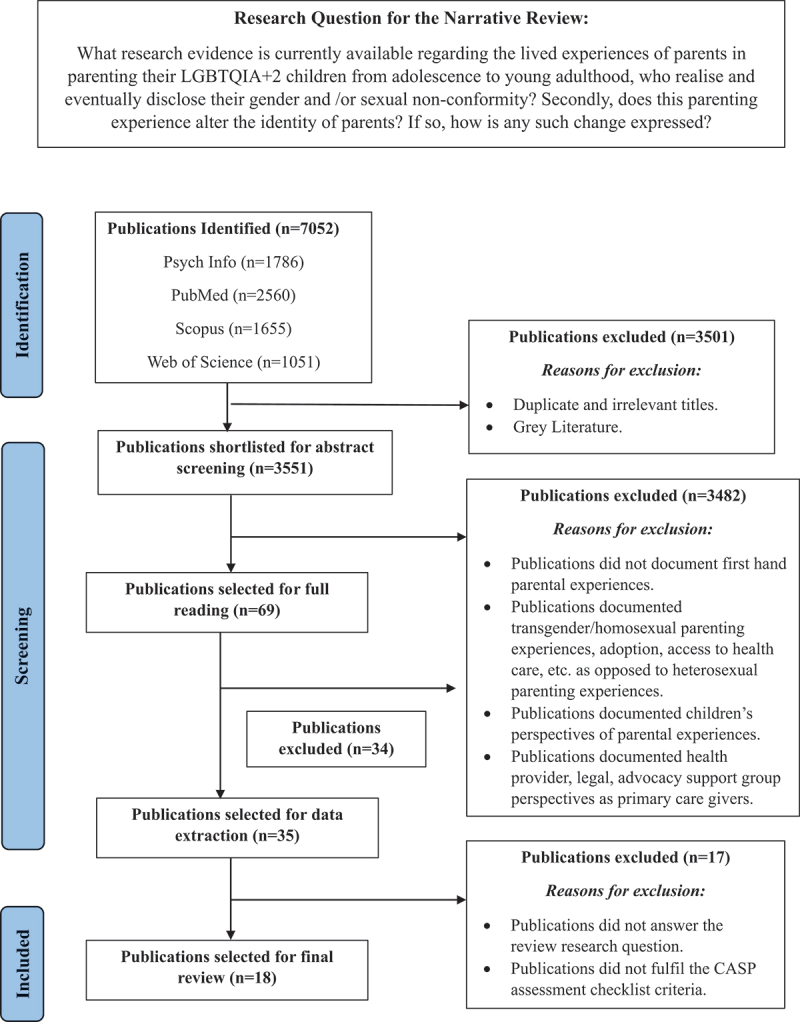
Flowchart of studies selected for the narrative review on the parental narratives of parenting a LGBTQIA+2 child. Flowchart conceptualised according to the PRISMA guidelines as specified by page et. al. And adapted from Abreu (2022).

Search keywords included parenting, parental experiences, identity, parental narratives, gender minority, gay, homosexuals, gender variance, gender dysphoria, LGB, LGBT, parent–child relationships, disclosure, sexual minority youth, fathers, mothers, parent support, parent adjustment, parental identity, etc. All terms were used in combination using AND and OR as separators. A literature search was conducted from April 2023 to November 2023 in PUBMED, Scopus, Web of Science and Psych Info through the Symbiosis International University Central Library system to select articles that fulfilled the criteria of the research question (Figure 2). The search was limited to the last 23 years (i.e., 2000–2023) and included full text, open access, journal articles published in English. The PICO framework was applied to the search strategy to include articles about first-hand experiences of parenting a LGBTQIA + 2 child. Articles were sifted for subsequent reading of titles and review of abstracts by the authors. Only studies that documented first hand parental experiences of parenting their child from the onset of gender and/or sexual non-conformity in adolescence to young adulthood were selected for full reading. Qualitative, quantitative, and/or mixed methods studies documenting parental sentiments were included.

#### Defining information extracted from the selected studies

The data extraction process was adapted from Lockwood et al., 2020 in (Abreu et al., 2022). This included name of author(s); year and journal of publication; objective, place, participants, data analysis, main results, and limitations of the study. Data extraction followed the PICO strategy in extracting data about parents, their parenting experiences, and their emotional transition to depict their identity change.

#### Assessing studies included in the review

The articles shortlisted for full reading and subsequent data extraction were further assessed for quality and methodological robustness using the 10-item CASP checklist (CASP Qualitative Checklist 2018) (Table A2 in Appendix A). Studies were assessed for their validity and authenticity of the results. All studies validated through the CASP assessment process and fulfilling the research question were included in the final review including one systematic review that was assessed using the CASP systematic review checklist (Table A1 in Appendix A) (CASP Systematic Review Checklist 2018).

#### Interpretation of results and synthesis of knowledge using IPT

IPT proposes that parents experience a change in their identity following their child’s disclosure (G. Breakwell, 2015; Jaspal, 2020). The studies in this review documented parental experiences at and after disclosure. These studies were reviewed using the IPT as a theoretical framework to trace the change in parental identity. (*Refer* Figure 1). The quotations used in the review papers were analysed using the four phases, seven motives and three interactions of the IPT model. Firstly, parental quotations were classified into assimilation, accommodation, adjustment and evaluation phases depending on the parenting phase described in the data excerpts cited within each paper. Secondly, parental expressions were then labelled with the motives and interactions of the IPT, using the definitions. These motives and interactions were then classified into the four phases of the IPT model. This process revealed the motives behind the identity change during each phase of the IPT and what parental interactions transpired during each phase. Parental emotions expressed in the quotations were also classified across the four phases of the IPT to track the change in parental identity through emotions. Since the studies in the review included accepting heterosexual or cisgender parents from LGBTQIA + 2 support groups, willing to be a part of the research, all the components of the IPT, especially the evaluation phase of acceptance were easy to categorize. However, this evaluation phase may not be so evident in a dataset where non-accepting parents might be included.

#### Ethical Consideration

Since this was a narrative review and did not directly involve human study participants, no ethical clearance was required.

#### Researcher Positionality

The authors of this paper identify as heterosexual females and have researched the phenomenon as outsiders, cognizant of their individual perspectives and research knowledge of parenthood. Two of the authors are of Indian origin and one author is of English origin.

##### 2.3. Definitions used for the review

## 3. Results

The study selection process is detailed in Figure 2. Eighteen articles were finalized for review and data synthesis. All articles (100%) were published in English. The majority of shortlisted articles (78%) were published between 2011 and 2023 (Table 2). Twenty-two percent of the articles were published between 2000 and 2010. Majority of the included studies (78%) were based in the US, the remaining 22% were based in South America, United Kingdom, Serbia, and New Zealand (Table 2).

**Table 2.** Data extraction sheet of place, year of study, study objectives, methods, main results, and limitations of the shortlisted papers for the narrative review on parental narratives of parenting a LGBTQIA + 2 child. Adapted from Abreu et al. (2022)..

Sr.No.	Author/Journal/ Year/Country	Study Objectives	Study Sites/Participants	Study Methods	Main Results/Observations	Limitations of the Study
1.	Gray et.al./Family Process/2016/USA.	- The experience of parenting a GV child and the mutual influence between the child, family, and environment	- America. - 11 parents.	- Qualitative analysis of semi-structured interviews. - Grounded theory approach.	- Parents rescued and protected their child from gender variance. - Parents embraced their child’s gender variance and advocated for a more accepting and tolerant world.	- Racially and economically similar sample. - Parents were connected to a GV affirmative source. - No exploration of child factors, such as age and natal sex, impacting parents’ experiences.
2.	Grafsky/JGLBT Family Studies/2014/USA.	- Parental perspectives of disclosure of SMY aged 14–21 years.	- America. - 8 parents.	- Semi-structured interviews. - Parent Child Closeness inventory. - NVivo 9.0. - Constructivist grounded and symbolic interaction.	- “Becoming” a parent of an LGB child conceptualizes parental experience. - Parents understand and re-envision parenting.	- Uniracial, small sample from a common recruitment place.
3.	Gattamorta et.al./Journal of GLBT Family Studies/2019/USA.	- Explore the impact of having a child come out as LGB.	- South Florida. - 10 Hispanic parents.	- In-depth parental experiences. - Grounded theory. - Nvivo 11.	- Initial negative reactions were followed by a stressful parent-child dynamic, followed by an eventual acceptance and improved parent-child relationship.	- Small sample size. - Mostly female participants. - Acceptance process not defined.
4.	Newcomb et.al/Sexuality Research and Social Policy/2018/USA.	- Understand parental communication, knowledge, monitoring of healthy sexuality, dating and sexual behaviour of their LGBTQ adolescents.	- USA. - 44 parents.	- Online FGDs. - Inductive and deductive coding. - Dedoose.	- Parents of LGBTQ adolescents require education and support to help their children have positive sexual health outcomes.	- Accepting mothers. - White participants.
5.	Saltzburg S/Social Work/2004/USA	-Phenomenological study of assigning meaning to the child’s disclosure and redefining post-disclosure parenting.	- New England, USA. - 7 parents	- In depth interviews - Social constructionist lens.	- Parental inkling of the child’s non-conformity becomes certain after disclosure. - Initial emotional detachment and fear of estrangement is followed by adjustment.	- Racially and socio-economically non-representative sample.
6.	Littman L/PLOS ONE/2018/USA.	- Parents’ perspectives and factors contributing to the onset and/or expression of gender dysphoria in their AYA children	- America - 256 parents.	- Online survey and open-ended questions. - Frequencies, percentages, ranges, means and/or medians. - Grounded Theory.	- Rapid-onset gender dysphoria has not yet been clinically validated. - Social media may influence maladaptive coping mechanisms in children.	- Parental coping strategies and mental well-being not explored. - Parent child dyads not considered for data collection.
7.	Jaspal, R./Journal of GLBT Family Studies/2019/UK.	- Parental perspectives of identity, well-being, and coping.	- UK. - 12 British South Asian parents	- Unstructured interviews - Inductive thematic analysis using IPT.	- Self-esteem and identity continuity is threatened. - Parents may resort to denial and social isolation.	- Parental reactions of other sexual and ethnic minority groups not considered.
8.	Larson J/The Annals of Family Medicine/2021/USA.	- Documents first-hand experience of parenting a trans man child.	- US - 1 parent	- Reflective paper.	- Parents need to be receptive to their child’s needs. - Extensive research about parental perspectives needs to be conducted.	- Perspective from an educated, well-informed, White, accepting parent.
9.	Kidd K.M., et.al./Journal of Adolescent Health/2021/USA.	- Parent and caregiver perspectives on the legislation against gender affirmation treatment and perceived effects on TGDY’s mental health.	- 43 US states - 273.	- Online Survey of mixed questions. - Quantitative analysis using Stata 15.1. - Qualitative inductive thematic analysis.	- Parents feared the laws would worsen their child’s mental health. - Parents feared discrimination, loss of access to gender-affirming medical interventions, and autonomy of medical decision-making.	- US based study of affirming parents.
10.	Mirković, V., & Jerković, I./The Qualitative Report/2021/Serbia.	- The maternal experiences of having LGBTQ children.	- Serbia. - 8 mothers.	- Interpretative qualitative phenomenological analysis.	- Disclosure is followed by confusion and an eventual adaptation that forms the family identity.	- Only mothers’ perspectives were explored.
11.	Horn, A. J., & Wong, Y. J./Psychology of Men & Masculinity/206/(2016).	- The positive aspects of the relationship between young gay men and their fathers.	- USA. -5 fathers.	- Unstructured interviews.	- Fathers loved their sons, viewed their relationship as both changed and unchanged after disclosure. - Fathers valued a deep connection to their sons and were personally and positively changed. - Fathers varied in how and from whom they sought support.	- Small sample size of White, accepting fathers.
12.	Spivey et.al.,/American Psychological Association/2018/USA.	- Examine parent and child characteristics on how parents respond, or would respond, to gender nonconforming behaviours.	- US -236 parents.	- Online mixed survey. - Multivariate and bivariate analysis. - Thematic analysis.	- Parents are uncomfortable with their child’s gender-nonconformity and intervene to meet societal expectations. - This behaviour was especially significant for gender non-conformity in boys.	- Homogeneous sample. - Non-representative of the racial, ethnic, and religious diversity.
13.	Kristopher M. Goodrich, Dennis D. Gilbride/Journal of LGBT issues in counselling/2010/USA.	- Refining and validating an emergent theory of family functioning.	- USA -687 parents.	- Multiple regression analysis. - AMOS 16	- Parents’ preconceived notions, needs, initial emotional reaction, religiosity, perceived level of social support, cognitive flexibility, empathy, and family’s behavioural response to the child’s disclosure play a role in a parent’s adjustment to the child’s disclosure.	- White, well-educated, middle-aged, middle to upper-middle class, liberal, biological mothers. - Study inclines toward parents who have gone through the process than those currently going through it. - Unable to show a causal relationship
14.	Goodrich, Kristopher M./Journal of LGBT Issues in Counselling/2009/USA.	- Explore experiences of families whose adult sons or daughters identify as LGB.	- USA -13 parents.	- Qualitative study - Grounded theory.	- Parents perceived their LGB child defined them successful as described by the emergent model for parent success.	- Lack of ethnic, sexual and gender diversity in the sample.
15.	Mary Jane Phillips, et.al.,/Journal of LGBT Issues in Counselling/2008/USA.	- Develop a model of parents’ adaption to their child’s disclosure. - Explore parental understanding of their changes as they adjust with their children’s disclosure.	- South Eastern USA. -17 parents.	- Semi structured interviews. - Thematically analysed. - N6 software.	- Culture plays a differential role in the parental identity developed through early, middle, and later parental adjustments post disclosure. - These include emotional and religious dimensions.	- Uniracial sample of accepting parents.
16.	Dangaltcheva A, Booth C and Moretti MM/Youth. Front. Psychol./2021/	- Create a new programme, for caregivers of transgender and gender non-conforming youth.	- USA -20 parents.	- The programme was video recorded and transcribed. - The transcription focused on parents’ reflections related to gender, youth mental health, and the parent-teen relationship.	- Parents learnt about parent-attachment that enhanced their understanding of their teen’s gender journey. - Parents understood their journey as a parent.	- US based sample of accepting parents.
17.	Julia de Bres & Ia Morrison-Young/LGBTQ+ Family: An Interdisciplinary Journal/2023.	- Describe the discourses parents adopt to support their transgender children.	- New Zealand. -20 parents.	- Use of visual art. - Unstructured interviews.	- Visual metaphors represented family resilience, personal transformation, shifting gender ideologies, depathologisation, child-led parenting, unconditional love, protection, and uncertainty about the child’s future.	Children’s perspectives were not included in the study.
18.	Daniella de Abreu et.al.,/International Journal of Environmental Research and Public Health/2022/South America.	- Reviewing the dynamics of primary social networks to support mothers, fathers, or guardians of transgender children and adolescents.	- Researchers based in South America.	- A systematic review of 21 qualitative studies using the PRISMA guidelines. - Derive parental perspectives of their social networks.	- Parental primary support systems were fragile and conflicting. - Parents experienced strained marriages and friendships and are treated with hostility and harassment.	- Grey literature not included.

### 3.1. Methodological considerations of included papers

All studies included in the review presented valid results using a robust methodology with viable future and local implications (*See* Tables AI and AII *in* Appendix A). Seventy-eight percent of the studies were qualitative in nature, 17% employed mixed methods and 5% were quantitative studies. All studies were justified in their ethical approach, methodology, research design, data collection and analysis. However, 22% of these studies (Dangaltcheva et al., 2021; Gattamorta et al., 2019; Littman & Romer, 2018; Newcomb et al., 2018) failed to consider the researcher and participant relationship. The researcher–participant relationship was not applicable for one review (Abreu et al., 2022) and one theoretical paper (Goodrich & Gilbride, 2010). One paper (Larson, 2021) was a first-hand parental experience and did not follow the conventional research methodology but was included in the review since it fulfilled our main review research question.

### 3.2. Limitations of the reviewed studies

Majority of the reviewed studies (72%) were conducted in the US, this meant the participants included in these papers skewed the reported outcomes towards a White perspective. Seventy-seven percent of these studies reported having a small, uniracial, sample of affirming and accepting parents affiliated to a LGBTQIA + 2 support group as their major limitation. Twenty-two percent of the studies reported having a non-gender diverse sample, including mainly mothers, or mostly fathers, or parents of only one gender or sexual minority, participating in the study as their limitations. One Hispanic study (Gattamorta et al., 2019) observed that the process by which parents eventually accepted their child was not clearly explained. Another study (Littman & Romer, 2018) found that parental coping mechanisms, methods of coping mechanisms and parental mental well-being were not explored. Two studies (De Bres & Morrison-Young, 2023; Littman & Romer, 2018) commented that their inability to collect qualitative data from parent–child dyads was a limitation to their research. One study (Goodrich & Gilbride, 2010) highlighted the need of studying generational difference in parents, where experiences of older parents, who have already experienced the process of parenting their child, were not compared with younger parents who may still be experiencing the process. Meanwhile, Gray et al. (2016) noted that they did not explore if children’s factors, such as age and natal sex, may have impacted parents’ experience. Only one study (De Bres & Morrison-Young, 2023) explicitly reported the gender and sexual identity of the participating parents. This influences the findings from the reviewed studies from a heteronormative and cisgender perspective (Table 2).

## 4. Discussion

### 4.1. Parental emotions, motives, and interactions in the assimilation phase of IPT

**Figure 3.**
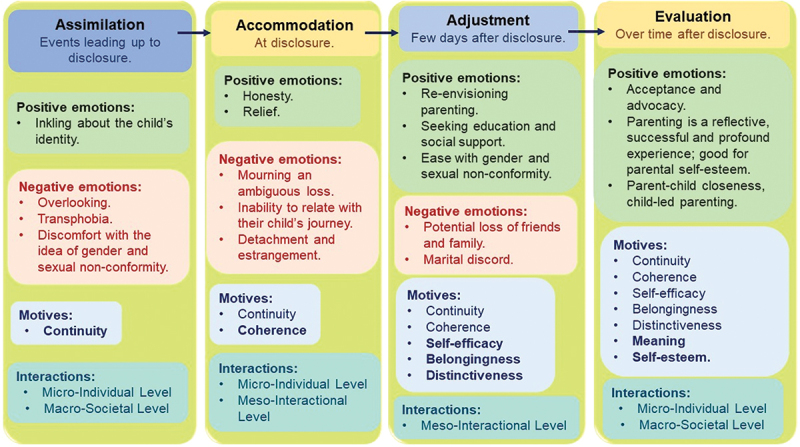
Parental emotions, motives and interactions across the phases of the identity process theory framework.

The assimilation phase entails the absorption of information that acts as a potential precursor for the change in parental identity. In the assimilation phase, parents are motivated to continue their original parental identity, thus influencing parental reflective and social interactions *(Refer* Figure 3) (Bardi et al., 2014; G. M. Breakwell, 2014; Chryssochoou, 2014; Sablonnière & Usborne, 2014). The assimilation phase of the identity process is an escalation towards the main event that will eventually initiate an identity change, which in this case, is disclosure. Parental emotions during assimilation include having an inkling or doubt about their child’s non-conformity to gender and/or sexuality (*Refer* Figure 3). Parental inkling stems from childhood behaviours like gender non-conforming external appearances and mannerisms, choice of toys or magazines, preferences for same-sex friendships and extreme reactions to family discussions about gender and sexuality, etc. (Grafsky, 2014; Littman & Romer, 2018; Saltzburg, 2004). For example, parental comments from the reviewed studies include statements as:

*“I used to buy her ‘girlie’ things and she used to push them away* (Grafsky, 2014).”

*We always knew. As the Christmases progressed … . buy the little dolls and the trucks, and he would open the male toy and put it down and grab his sister’s toys … … .I remember thinking this is not okay. … Perhaps in the very back of my mind there was always that wonder, “Is he gay?”* (Mother J) in (Saltzburg, 2004).

Another parent in (Saltzburg, 2004) states, *“ … … ., I went up to his room one day and … .those Teen Beat magazines … have pictures of the boys? The whole wall was filled with … —cute boys. I mean, I am thinking, “He should have pictures of girls on the wall!” I knew then. I did not want to really know. I hoped against hope it would not be, but in my heart, I knew then. It was like the photos on the wall were the “writing on the wall.”* (Mother P).

*“In general, cis-gendered people are considered evil and unsupportive, regardless of their actual views on the topic. To be heterosexual, comfortable with the gender you were assigned at birth, and non-minority places you in the ‘most evil’ of categories …”*(Littman & Romer, 2018)

Parents’ silence around their child’s non-conforming behaviour in the assimilation phase is often motivated by the parental desire to continue and maintain the original parental identity (Sablonnière & Usborne, 2014). These parental identity behaviours influence parental micro-individual and macro-social interactions. The micro-individual interactions are reflective and internal in nature, whereas the macro-social occur within the social and family clusters in the existing identity (Chryssochoou, 2014). In the micro-individual interactions, parents avoid confiding in anyone about the behavioural changes they notice in their child and many prefer keeping their observations to themselves (Bardi et al., 2014; Sablonnière & Usborne, 2014). The phrases *“In the back of my mind,” “hoped against hope”* “*I did not want to really know,” “thinking this is not okay,” “Perhaps in the very back of my mind there was always that wonder”* in the above quotes reflect this internalization. In order to conserve their original parental identity, parents may continue interactions with their extended familial and social groups without bringing up the changes they observe in their child (Spini & Jopp, 2014). It may be hypothesized that parents use the macro-social interactions to mask their internalized reflections about their child.

### 4.2. Parental emotions, motives, and interactions in the accommodation phase of IPT

The accommodation phase of the IPT entails creating space for a new piece of information that may confirm the identity change (G. M. Breakwell, 2014; Sablonnière & Usborne, 2014). In this context, this accommodation stage involves the event of disclosure. The identity change in assimilation phase is motivated by the continuity and coherence principles *(Refer* Figure 3) (Bardi et al., 2014; Sablonnière & Usborne, 2014). Coherence ensures that parents balance their other existing identities of being a husband, wife, or parent of their other heterosexual child along with their new found identity as the parent of a LGBTQIA + 2 child. The identity change in this phase can drive both the micro-individual and meso-interactions *(Refer* Figure 3) (Chryssochoou, 2014).

At the disclosure stage, many parents experience varied emotions as they are confronted with the reality of their child’s non-conformity to the “customary” gender and sexual orientation norms. It is at this point that parents start parenting a LGBTQIA + 2 child and the process of identity change is initiated and confirmed (Grafsky, 2014). Although parents appreciate the honesty of their child and are relieved at the revelation of an information they sought to hear for long, parents may also experience emotions such as shock, grief, fear, concern, loss, stress, confusion, etc. as they mourn an ambiguous loss and struggle to identify with their child’s transition. (*Refer* Figure 3). These emotions stem from uncertainty, concern, religious and social beliefs which form the parental identity (Gray et al., 2016). As Newcomb *et al*. discuss,

Parents described their experiences at disclosure as, *“I felt devastated…I was petrified for his future, for being ostracized possibly, disliked, hated or worse hurt by someone who doesn’t understand…I think it was partly my expectations held for the future and partly because it made me extremely sad that this could have happened to him, born with the wrong body parts. We said some pretty stupid, naive things to him back then”* (Newcomb et al., 2018).

Meanwhile, other parents may express relief in learning more about their child since intuitively they already knew about their child’s non-conformity and were glad the truth was now out in the open (De Bres & Morrison-Young, 2023; Goodrich, 2009; Goodrich & Gilbride, 2010). Parents observed that having a doubt about their child before the disclosure helped them examine their emotional reactions at disclosure and find ways of supporting their child better (Horn & Wong, 2016). For instance, as Gattamorta and colleagues observe,

One parent stated, *“In some ways it was a relief, his coming out was, to us, was very, very atypical in that when he came out to us, we said, ‘yeah, we knew.’ … We were just kind of waiting for about 10 years for this to happen”* (Gattamorta et al., 2019).

Experiencing relief at disclosure is motivated by parents’ understandable desire to continue parenting their child. However, parents must also maintain a balance between their identities of parenting a non-conforming child along with being a husband, wife, a working professional, parenting a heterosexual child, etc.

For example, as (Jaspal, 2020) documented a parental narrative in their research study;

“*I just thought I had failed as a mother and failed in my duty as a good Muslim. It had a bad impact on me. I felt disgusted with him and also with myself. And I was alone in this feeling because I couldn’t tell my husband anything or my other children even”.*

The mother in the above quotation was trying to continue her identity as a mother to her gay son, while trying to be “*good Muslim*,” along with being a “*wife”* to her husband and *“mother”* to her other heterosexual children.

Since parents are still navigating their own new-found identities in this accommodation phase, many report choosing to keep parental reflections to themselves as is evident in the mother’s dilemma above, when she expresses “*being alone”* in the process. This “loneliness” in the parental journey becomes a driver for parental meso-interactions. Parents want to be a part of social groups who experience and share their dilemma. The meso-interactions are stimulated by the new found identity where parents are experiencing an existential aloneness and want to solicit solidarity of like-minded counterparts (Pehrson & Reicher, 2014).

As one of (Saltzburg, 2004) the parents states:, “*I wanted to cry with other parents feeling like me.”*

While trying to accommodate the event of disclosure in their lives, parents seek support in similar counterparts as they strive to continue and balance their new parental identities.

### 4.3. Parental emotions, motives and interactions in the adjustment phase of IPT

The adjustment phase of IPT occurs a few days after disclosure. Parents begin to better understand their role in the child’s journey and adjust themselves to their new parental identity. Identity change in the adjustment phase is motivated by self-efficacy, belongingness, and distinctiveness along with the previous motives of continuity and coherence *(Refer* Figure 3) (G. M. Breakwell, 2014; Rusi et al., 2016). Parents attempt to be confident, effective and in control of their new identity. Parents maintain closeness to their child and advocate for acceptance from others as they embrace their uniqueness as parents (Dixon et al., 2014). The identity interactions in this phase are purely meso-interactional in nature, *(Refer* Figure 3) where parents interact with groups that are shaped by their new identity (Sablonnière & Usborne, 2014). In this phase, parents re-envision parenting. They educate themselves by seeking community resources and social support. They attempt to get comfortable with gender and/or sexual non-conformity *(Refer* Figure 3) (Goodrich, 2009; Larson, 2021; Martin et al., 2010; Phillips & Ancis, 2008).

For example, “*Once I finally realized that my son was gonna be safe, I got that out of my system … … …”*(Grafsky, 2014).

As one parent from (De Bres & Morrison-Young, 2023) stated*“ … … .We need to research this’*. They extensively researched transgender matters. They incorporated their findings into regular conversations with their extended family by saying, *“this is the research we’ve read; these are the doctors we’ve seen; this is how we’re going to parent our child, and you might want to watch this documentary”.*

Parents also devised mechanisms of disclosing their child’s identity to the extended social circle, as a means of protecting their child. This established parental control and confidence in the uniqueness of the new parental identity (Gattamorta et al., 2019; Gray et al., 2016; Horn & Wong, 2016).

As parents from the above studies observed:

*“We made a deal that he will tell his friends and that I will tell the family friends and family” (*Gattamorta et al., 2019).

*“At work at first, I couldn’t, I didn’t say anything. And then I would hear the jokes about the gays and things like that. And after a while I started, when I would hear them telling a gay joke, I would walk over and when everybody started to laugh, I would say, ‘What’s so funny about that?’ and I would just kill their punch line. And that was kind of my way of starting to come out at work.”* (Horn & Wong, 2016).

However, in this adjustment phase many parents may also experience a potential loss of friends, experience harassment and hostility from relatives and in some cases, marital discord (Abreu et al., 2022). Parents may also fear being reported to the “authorities” for not being supportive of their child’s choice or even indulging too much in alternative social norms *(Refer* Figure 3). These isolating social factors initiate the meso interactions where parents seek to adjust by actively engaging with parental LGBTQIA + 2 support groups (Pehrson & Reicher, 2014).

As one parent from the reviewed papers stated: *“So many people come through PFLAG … … Many of those folks couldn’t readily accept their child and support them, and in fact, [my son’s] father could not. … … … .”*. (Phillips & Ancis, 2008)

*(PFLAG is Parents, Families and Friends of Lesbians and Gays)*

The involvement of parental support groups can offer parents an opportunity to interact with like-minded people while continuing to be effectual in parenting their LGBTQIAI + 2 child (Bardi et al., 2014). These identity driven meso-interactions offer parents a sense of belonging and distinctiveness as parents of a LGBTQIA + 2 child, *(Refer* Figure 3) thus instilling confidence, and a sense of adjustment for the new parental identity (G. M. Breakwell, 2014).

### 4.4. Parental emotions, motives and interactions in the evaluation phase of IPT

The evaluation phase is the last phase of the IPT and marks the completion of parental identity change (G. M. Breakwell, 2014; Jaspal, 2014). The parental emotions and interactions in this phase are motivated by all the seven identity motives of the IPT (Sablonnière & Usborne, 2014). In this phase, parents have found meaning in their new identity and consider parenting a LGBTQIA + 2 child, a matter of self-esteem *(Refer* Figure 3). Parents are also able to continue, be effective, maintain compatibility, distinctiveness and belonging towards their new parental identity (Spini & Jopp, 2014). The interactions in this phase are similar to the assimilation phase and include the reflective micro-individual and macro-social *(Refer* Figure 3) (Pehrson & Reicher, 2014).

Parental emotions in evaluation phase are of growing acceptance (Amiot & Rusi, 2014) *(Refer* Figure 3). Parents may, as part of their own development, begin to re-evaluate themselves and reflect on what it means to be a parent to a LGBTQIA + 2 child. They can articulate their acceptance and learn to embrace gender and/or sexual variance. Many parents begin to consider parenting a LGBTQIA + 2 child as a rewarding, privileged experience, beneficial for their self-esteem and personal growth. They reflect on the parenting experience as an intense, profound, and awakening experience that has made them generous, tolerant and emphatic *(Refer* Figure 3). Parents evaluate their parenting as successful and experience an improved closeness with their child. They also sense a change in their parenting style which is less directive (De Bres & Morrison-Young, 2023; Flores et al., 2021; Grafsky, 2014; Katz-Wise et al., 2016; Larson, 2021; Mills-Koonce et al., 2018; Mirković & Jerković, 2021; Rosati et al., 2020; Thornburgh et al., 2020; Van Bergen et al., 2021).

As one parent states in a study: *“It has made me a better person—I definitely feel like I’m so lucky to have a trans child”*. (De Bres & Morrison-Young, 2023).

At the micro individual level, parents are constantly evaluating themselves and reflecting on their parenting style and strategy. On a macro-societal interactive level, they interact with their extended social and familial groups within their new identity. As they gain more comfort in their new found identity and role, they advocate for acceptance and protection for their child (Chryssochoou, 2014; Marková, 2007; Pehrson & Reicher, 2014).

As one parent reminisces, “*I think one of the things that was very helpful for me was that I devoted some of my professional energies to the issue of sexual minorities … and started networking to get to know the LGB, … … … . … … . and that was very, very helpful to get their perspective because I could relate to them on a professional level and to see really what they had been, gone through.”* (Mirković & Jerković, 2021).

As a part of the macro-societal interactions, parents are also able to acknowledge their children’s gay partners as a part of their family (Chryssochoou, 2014; Horn & Wong, 2016).

*“Now I gotta start dealing with it. If he wants to bring [her son’s boyfriend] around family events and things like that, well then, we’re gonna have to tell the family, because I’m not gonna exclude him. And then I got to the point where, if people weren’t gonna accept him, then they were not gonna be in my life. I made a decision, if anybody gives me any type of negative feedback or you know, says bad things or don’t want him around or don’t wanna accept him then, I’m done with them. I drew a line*” (Grafsky, 2014).

The evaluation phase of the IPT witnesses solely positive parental emotions (*Refer* Figure 3). The amalgamation of all the identity motives and the return to their initial micro-individual and macro-societal interactions prove that although parents undergo various emotional and identity phases, they are able to restore their initial interactions in their new-found identity (Marková, 2007; Sablonnière & Usborne, 2014). It may be postulated that the meso-interactions in the accommodation and adjustment phases of the IPT aid in re-instating the previous micro and macro interactions of the assimilation phase in the evaluation phase of the IPT. Another remarkable observation is that it takes all the seven identity motives of the IPT to complete the identity process change culminating in the evaluation phase which marks the eventual acceptance of the child. Throughout the identity change process, parents strive to maintain and augment their role as care givers and nurturers in their old as well as new-found parental identity (Amiot & Rusi, 2014).

### 4.5. Strengths and limitations of the review

This review successfully highlights the process of change in parental identity through the IPT by employing quotations from previously published studies. However, we suggest that a more complete dataset of findings from future research may provide deeper insight into the parental identity change process and thus yield varied results. The focus of this review was not to highlight the mental well-being of parents while parenting a LGBTQIA + 2 child. From the retrieved literature considered, we were unable to elucidate the effect of identity change process on parental mental health and well-being, thus being only partially able to answer the question: What is driving what? Although parental well-being has received much academic and research attention, we found this aspect to be less well prioritized in the studies we were able to include for the review, given the critical appraisal process adopted (Mehus et al., 2017; Vázquez et al., 2023; Zhou et al., 2021). When parental identity is thus threatened in society, it may also have mental health implications (Loewenthal, 2014). However, due to the current research we were able to include, the phenomenon of threat to identity was not explored in any depth within this review. We suggest this might constitute a separate, if related, topic for future research. Our rationale here was that the perceived threat to identity has, to date, been mainly studied in the more conservative “traditional” Asian contexts (Jaspal, 2020; Loewenthal, 2014). In such settings, parental pride is equated with one’s cultural identity and social standing. A child’s non-conformity threatens the parent’s social identity of respect and self-esteem, thus affecting parental mental health.

The review does not comment on the gender and/or sexual identity of parents. This is attributed to the lack of evidence we were able to identify on parental gender and/or sexual identity in the studies included in our review. Although the identity of the parent may affect their reaction to their child’s disclosure, this aspect has not been discussed in detail in this review.

Our review therefore does not explore the parenting phenomenon from other framework analyses which may yield varied results, such as the commonly used Adjustment to Change Theory which contributes to the universal theory of change phenomenon. Instead, we were interested in exploring the literature from the more innovative approach of using the IPT perspective. We acknowledge that limiting our review to examining the evidence from a single framework runs the risk of viewing the phenomenon from a single perspective, however we were interested in how this approach may affect the context of identity. Moreover, Adjustment to Change Theory has also been directly used to study adjustment in the LGBTQIA + 2 population (Umaña-Taylor, 2023) and in the non-LGBTQIA +2 context used to study institutional adjustment (Troub, 1983). Identity in society is thus an evolving phenomenon and there will always be additional principles that could be employed to deliver further, deeper, insights into the identity phenomenon. However, we suggest that the four phases of IPT from assimilation to evaluation are explained in detail in this review, supported by the evidence obtained from the papers we were able to include.

We were more interested in identifying the current evidence in our review that included studies of “accepting” parents who were part of a support group. These parents recounted their parenting journey: from the evidence we found that this culminated into coming to terms with, and accepting, their child’s “newer identity”. However, a heterogeneous sample of parents who have not come to terms with their child’s non-conformity may yield different outcomes, and offer additional insights into the experience of parental identity. Thus, this review did not consider the influence of parental gender and sexual identity on their parenting experiences. Additionally, since the majority of studies included in the review did not document parent identity, we were not able to examine in any detail the phenomenon of transgender parenting. We were also unable to consider any variance in identity change between mothers and fathers. Since this was not an original dataset, dissecting these differences was a challenge as most studies which met our careful selection and criteria for inclusion did not have equal numbers of father and mother participants.

## 5. Conclusions

This review offers additional insights that present evidence for the process of identity change that parents undergo after their child’s disclosure, and that can be helpfully explained through use of the IPT model. We suggest this approach offers much potential for future research that could, for example, be employed on a more complete dataset to inform and confirm the stages of parental identity change we identify. This narrative synthesis of current available evidence emphasizes the need for studying parental experiences in other non-white conservative settings, especially among Asian cultures, where identity is a nuanced phenomenon layered with social and cultural connotations. Such identity motives drive parental emotions and the identity interactions throughout the IPT phases. These emotions and interactions form a period of considerable personal change development throughout the IPT phases, and may be a stressful time for such parents until later, more complete, acceptance at the evaluation phase of the IPT. We highlight parental well-being area as a necessary phenomenon of study if any society is to limit the intergenerational effect of mental health on sexual and gender minority populations. This review on parental transition advocates the need for further studies to examine parental well-being, especially in more conservative Asian settings where identity is equated with social standing and a child’s non-conformity may threaten parental identity with related mental health and well-being implications. We consider our review offers additional insight on parental perspectives that can inform and support health care providers, advocacy, and social groups to plan effective and sensitive intervention programmes for both core and peripheral LGBTQIA + 2 community.

In summary, the main implication of this review has been to offer further understanding of parental identity change and its possible adverse emotional effects on parents and children from assimilation to acceptance in the IPT evaluation phase. We recommend that there is an identified need to consider processes as to how we might reduce the acceptance time for both parents and their family. The relationship between mental health and change in parental identity needs to be explored across the phases of the IPT and, we consider, merits further research. This is to determine if the identity change is affecting and driving the parental mental well-being or vice versa. Tracking mental health transitions across the identity phases will aid the development of more specific mental health interventions that can be targeted across the parenting phase, to help strengthen parental identity motives, linked to the interactions of the IPT model.

## List of abbreviations

AYA: Adolescent Young Adults
CASP: Critical Appraisal Skills Programme
GV: Gender Variance/Variant
IPT: Identity Process Theory
LGBTQIA +2: Lesbian, Gay, Bisexual, Trans, Queer/Questioning, Intersex, Asexual, Two-spirited
PICO: Population, Intent, Context and Outcome
PRISMA: Preferred Reporting Items for Systematic Review and Meta-Analysis
SMY: Sexual Minority Youth
TGDY’s: Transgender and Gender-Diverse Youths

## Appendix A: CASP assessment of the selected studies

**Table AI.** CASP assessment of the review included in the narrative review on parental narratives of parenting a LGBTQIA + 2 child.

Name of the Study: Daniella de Abreu et.al., 2022			
**Section A**			
Did the review address a clearly focused question?			Yes
Did the authors look for the right type of papers?			Yes
Do you think all the important, relevant studies were included?			Yes
Did the review’ s authors do enough to assess quality of the included studies?			Yes
Did the review’ s authors do enough to assess quality of the included studies?			Yes
**Section B**			
What are the overall results of the review?	The review of 21 qualitative articles documents the familial support system available to parents of a gender variant child.		
What are the overall results of the review?	The results are coherent with previous findings.		
**Section C**			
Can the results be applied to the local population?		Yes	
Can the results be applied to the local population?		Yes	
Can the results be applied to the local population?		Yes

**Table A II.** CASP assessment checklist of papers for the narrative review on parental narratives of parenting a LGBTQIA + 2 child.

	Section A						Section B			Section C
	Are the results valid?						What are the results?			Will the results help locally?
**Study**	Was there a clear statement of the aims of the research?	Is a qualitative methodology appropriate?	Was the research design appropriate to address the aims of the research?	Was the recruitment strategy appropriate to the aims of the research?	Was the data collected in a way that addressed the research issue?	Has the relationship between researcher and participants been adequately considered?	Have ethical issues been taken into consideration?	Was the data analysis sufficiently rigorous?	Is there a clear statement of findings?	How valuable is the research?
Gray et al. (2016)	Yes	Yes	Yes	Yes	Yes	Yes	No	Yes	Yes	Presents themes of parental perspectives of across family and social interactions
Grafsky (2014)	Yes	Yes	Yes	Yes	Yes	Yes	Yes	Yes	Yes	Documents how parents re-envision parenting after disclosure.
Gattamorta et al. (2019)	Yes	Yes	Yes	Yes	Yes	No	Yes	Yes	Yes	Offers an ethnic parental perspective that explores cultural factors affecting parental experiences.
Newcomb et al. (2018)	Yes	Yes	Yes	Yes	Yes	No	Yes	Yes	Yes	Describes specific practices used by parents to prevent negative sexual health outcomes in their LGBTQ teens to inform the development of family-based sexual health programmes.
Saltzburg (2004)	Yes	Yes	Yes	Yes	Yes	Yes	Yes	Yes	Yes	It is a phenomenology study focusing specifically on the adolescent phase of parenting.
Littman and Romer (2018)	Yes	Yes	Yes	Yes	Yes	No	Yes	Yes	Yes	It explores the role and influence of social media on onset of gender dysphoria.
Jaspal (2020)	Yes	Yes	Yes	Yes	Yes	Yes	Yes	Yes	Yes	An UK based paper that documents perspectives of Muslim parents with gay sons using the Identity Process Theory as the framework to describe parental identity.
Justine Larson (2021)	NA	NA	NA	NA	NA	Yes	NA	NA	NA	This is a first-hand experience of a parent of a LGBTQIA + 2 child.
Kidd et al. (2021).	Yes	Yes	Yes	Yes	Yes	Yes	Yes	Yes	Yes	A post disclosure advocacy-oriented research focussing on child and parent autonomy.
Mirković and Jerković (2021)	Yes	Yes	Yes	Yes	Yes	Yes	Yes	Yes	Yes	An ethnic study exploring a new geographical area and an ethnic identity.
Horn and Wong (2016)	Yes	Yes	Yes	Yes	Yes	Yes	Yes	Yes	Yes	Solely documents paternal experiences as opposed to most studies with mothers as major participants.
Spivey et al. (2018)	Yes	NA	Yes	Yes	Yes	Yes	Yes	Yes	Yes	Differentiates parental experiences based on cultural, social, religious factors and especially gender of the child.
Goodrich and Gilbride (2010).	Yes	NA	Yes	Yes	Yes	NA	Yes	Yes	Yes	It validates the theory of family functioning after disclosure.
Goodrich (2009)	Yes	Yes	Yes	Yes	Yes	Yes	Yes	Yes	Yes	It discusses the parent identity in view of disclosure and following events
Phillips and Ancis (2008).	Yes	Yes	Yes	Yes	Yes	Yes	Yes	Yes	Yes	It explains the process of identity development of a parent from a parental perspective.
Dangaltcheva et al. (2021).	Yes	Yes	Yes	Yes	Yes	No	Yes	Yes	Yes	It explains the change in parent child relation trajectory.
Julia de Bres & Ia Morrison-Young	Yes	Yes	Yes	Yes	Yes	Yes	Yes	Yes	Yes	The study used a creative and novel method of art to express parenting experiences in a new geographic setting.

## References

[cit0001] Abreu, P. D. D., Andrade, R. L. D. P., Maza, I. L. D. S., Faria, M. G. B. F. D., Nogueira, J. D. A., & Monroe, A. A. (2022). Dynamics of primary social networks to support mothers, fathers, or guardians of transgender children and adolescents: A systematic review. *International Journal of Environmental Research and Public Health*, 19(13), 7941. 10.3390/ijerph19137941 35805599 PMC9265819

[cit0002] Amiot, C., & Rusi, J. (2014). *Identity integration, psychological coherence and identity threat: Linking identity process theory and notions of integration (chapter 8)—identity process theory*. https://www.cambridge.org/core/books/abs/identity-process-theory/identity-integration-psychological-coherence-and-identity-threat-linking-identity-process-theory-and-notions-of-integration/FA8151F77BE21C8EDB0ED8F6C579C51A

[cit0003] *Asexuality*, *Attraction*, and *Romantic Orientation*. (2021, July 1). LGBTQ Center. https://lgbtq.unc.edu/resources/exploring-identities/asexuality-attraction-and-romantic-orientation/

[cit0004] Ayhan, C. H. B., Bilgin, H., Uluman, O. T., Sukut, O., Yilmaz, S., & Buzlu, S. (2020). A systematic review of the discrimination against sexual and gender minority in health care settings. *International Journal of Health Services*, 50(1), 44–19. 10.1177/0020731419885093 31684808

[cit0005] Bardi, A., Jaspal, R., Polek, E., & Schwartz, S. (2014). Values and identity process theory: Theoretical integration and empirical interactions. *Identity Process Theory: Identity, Social Action and Social Change*, 175–200. 10.1017/CBO9781139136983.013

[cit0006] Bratt, A. S., Svensson, I., & Rusner, M. (2019). Finding confidence and inner trust as a parent: Experiences of group-based compassion-focused therapy for the parents of adolescents with mental health problems. *International Journal of Qualitative Studies on Health and Well-Being*, 14(1), 1684166. 10.1080/17482631.2019.1684166 31662062 PMC6830276

[cit0007] Breakwell, G. (2015). Identity process theory. In G. Sammut, E. Andreouli, G. Gaskell, & J. Valsiner (Eds.), *The Cambridge handbook of social representations* (1st ed. pp. 250–266). Cambridge University Press. 10.1017/CBO9781107323650.021

[cit0008] Breakwell, G. M. (2014). Identity process theory: Clarifications and elaborations. In G. M. Breakwell & R. Jaspal (Eds.), *Identity process theory: Identity, social action and social change* (pp. 20–38). Cambridge University Press. 10.1017/CBO9781139136983.004

[cit0009] Carnelley, K. B., Hepper, E. G., Hicks, C., & Turner, W. (2011). Perceived parental reactions to coming out, attachment, and romantic relationship views. *Attachment & Human Development*, 13(3), 217–236. 10.1080/14616734.2011.563828 21506028

[cit0010] *CASP Qualitative Checklist*. (2018). Retrieved July 14, 2023, from https://casp-uk.net/images/checklist/documents/CASP-Qualitative-Studies-Checklist/CASP-Qualitative-Checklist-2018_fillable_form.pdf

[cit0011] *CASP Systematic Review Checklist*. (2018). Retrieved July 14, 2023, from https://casp-uk.net/images/checklist/documents/CASP-Systematic-Review-Checklist/CASP-Systematic-Review-Checklist-2018_fillable-form.pdf

[cit0012] Chryssochoou, X. (2014). Identity processes in culturally diverse societies: How is cultural diversity reflected in the self? In *Identity process theory: Identity, social action and social change* (pp. 135–154). Cambridge University Press. 10.1017/CBO9781139136983.011

[cit0013] Coyle, A., & Murtagh, N. (2014). Qualitative approaches to research using identity process theory. In R. Jaspal & G. M. Breakwell (Eds.), *Identity process theory* (1st ed. pp. 41–64). Cambridge University Press. 10.1017/CBO9781139136983.006

[cit0014] Dangaltcheva, A., Booth, C., & Moretti, M. M. (2021). Transforming connections: A trauma-informed and attachment-based program to promote sensitive parenting of trans and gender non-conforming youth. *Frontiers in Psychology*, 12, 643823. 10.3389/fpsyg.2021.643823 34381395 PMC8350507

[cit0015] De Bres, J., & Morrison-Young, I. (2023). Storm clouds and rainbows: Visual metaphors of parents of transgender children in Aotearoa (New Zealand). *LGBTQ+ Family: An Interdisciplinary Journal*, 19(5), 382–404. 10.1080/27703371.2023.2231371

[cit0016] Dixon, J., Durrheim, K., & Masso, A. D. (2014). Places, identities and geopolitical change: Exploring the strengths and limits of identity process theory. In G. M. Breakwell & R. Jaspal (Eds.), *Identity process theory: Identity, social action and social change* (pp. 270–294). Cambridge University Press. 10.1017/CBO9781139136983.018

[cit0017] Flores, D. D., Greene, M. Z., & Taggart, T. (2021). Parent-child sex communication prompts, approaches, reactions, and functions according to gay, bisexual, and queer sons. *International Journal of Environmental Research and Public Health*, 19(1), 74. 10.3390/ijerph19010074 35010332 PMC8751024

[cit0018] Gattamorta, K. A., Salerno, J., & Quidley-Rodriguez, N. (2019). Hispanic parental experiences of learning a child identifies as a sexual minority. *Journal of GLBT Family Studies*, 15(2), 151–164. 10.1080/1550428X.2018.1518740 31440120 PMC6706085

[cit0019] Goodrich, K. M. (2009). Mom and dad come out: The process of identifying as a heterosexual parent with a Lesbian, gay, or bisexual child. *Journal of LGBT Issues in Counseling*, 3(1), 37–61. 10.1080/15538600902754478

[cit0020] Goodrich, K. M., & Gilbride, D. D. (2010). The refinement and validation of a Model of family functioning after Child’s disclosure as lesbian, gay, or bisexual. *Journal of LGBT Issues in Counseling*, 4(2), 92–121. 10.1080/15538605.2010.483575

[cit0021] Grafsky, E. L. (2014). Becoming the parent of a GLB son or daughter. *Journal of GLBT Family Studies*, 10(1–2), 36–57. 10.1080/1550428X.2014.857240 25685111 PMC4327774

[cit0022] Gray, S. A. O., Sweeney, K. K., Randazzo, R., & Levitt, H. M. (2016). “Am I doing the right thing?”: Pathways to parenting a gender variant child. *Family Process*, 55(1), 123–138. 10.1111/famp.12128 25639568 PMC5600542

[cit0023] Gyamerah, A. O., Collier, K. L., Reddy, V., & Sandfort, T. G. M. (2019). Sexuality disclosure among Black South African MSM and responses by family. *Journal of Sex Research*, 56(9), 1203–1218. 10.1080/00224499.2018.1559917 30633588 PMC6625940

[cit0024] Horn, A. J., & Wong, Y. J. (2016). Exploring the positive experiences of heterosexual fathers who parent gay sons: A phenomenological approach. *Psychology of Men & Masculinity*, 18(4), 268–279. 10.1037/men0000071

[cit0025] Jaspal, R. (2014). Social psychological debates about identity. In *Identity process theory: Identity, social action and social change* (pp. 3–19). Cambridge University Press. 10.1017/CBO9781139136983.003

[cit0026] Jaspal, R. (2020). Parental reactions to British South Asian young men who identify as gay. *Journal of GLBT Family Studies*, 16(4), 402–417. 10.1080/1550428X.2019.1684412

[cit0027] Katz-Wise, S. L., Rosario, M., & Tsappis, M. (2016). Lesbian, gay, bisexual, and transgender youth and family acceptance. *Pediatric Clinics of North America*, 63(6), 1011–1025. 10.1016/j.pcl.2016.07.005 27865331 PMC5127283

[cit0028] Kidd, K. M., Sequeira, G. M., Paglisotti, T., Katz-Wise, S. L., Kazmerski, T. M., Hillier, A., Miller, E., & Dowshen, N. (2021). “This could mean death for my child”: Parent perspectives on laws banning gender-affirming care for transgender adolescents. *Journal of Adolescent Health*, 68(6), 1082–1088. 10.1016/j.jadohealth.2020.09.010 PMC8041924 33067153

[cit0029] Larson, J. (2021). Parenting my transgender child: From loss to acceptance. *Annals of Family Medicine*, 19(6), 556–559. 10.1370/afm.2737 34750131 PMC8575513

[cit0030] Le, V., Arayasirikul, S., Chen, Y.-H., Jin, H., & Wilson, E. C. (2016). Types of social support and parental acceptance among transfemale youth and their impact on mental health, sexual debut, history of sex work and condomless anal intercourse. *Journal of the International AIDS Society*, 19(3S2), 20781. 10.7448/IAS.19.3.20781 27431467 PMC4949317

[cit0031] LGBT Pride Global Survey Report. (2021). Retrieved October 10, 2023, from https://www.ipsos.com/sites/default/files/ct/news/documents/2021-06/LGBT%20Pride%202021%20Global%20Survey%20Report_3.pdf

[cit0032] Littman, L., & Romer, D. (2018). Parent reports of adolescents and young adults perceived to show signs of a rapid onset of gender dysphoria. *PLOS ONE*, 13(8), e0202330. 10.1371/journal.pone.0202330 30114286 PMC6095578

[cit0033] Loewenthal, K. M. (2014). Religion, identity and mental health. In G. M. Breakwell & R. Jaspal (Eds.), *Identity process theory: Identity, social action and social change* (pp. 316–334). Cambridge University Press. 10.1017/CBO9781139136983.020

[cit0034] Manning, J. T., Fink, B., & Trivers, R. (2022). Parental income and the sexual behavior of their adult children: A trivers–Willard Perspective. *Evolutionary Psychology*, 20(4), 147470492211428. 10.1177/14747049221142858 PMC10303578 36503288

[cit0035] Marková, I. (2007). Social identities and social representations. In G. Moloney & I. Walker (Eds.), *Social representations and identity: Content, process, and Power* (pp. 215–236). Palgrave Macmillan US. 10.1057/9780230609181_12

[cit0036] Martin, K. A., Hutson, D. J., Kazyak, E., & Scherrer, K. S. (2010). Advice when children come out: The cultural “tool kits” of parents. *Journal of Family Issues*, 31(7), 960–991. 10.1177/0192513X09354454 20606708 PMC2893347

[cit0037] Matzner, S. (2022). Queer theory and ancient literature. *Oxford Classical Dictionary*. 10.1093/acrefore/9780199381135.013.8537

[cit0038] McGeough, B. L., & Sterzing, P. R. (2018). A systematic review of family victimization experiences among sexual minority youth. *The Journal of Primary Prevention*, 39(5), 491–528. 10.1007/s10935-018-0523-x 30206750 PMC6408293

[cit0039] Mehus, C. J., Watson, R. J., Eisenberg, M. E., Corliss, H. L., & Porta, C. M. (2017). Living as an LGBTQ adolescent and a Parent’s child: Overlapping or separate experiences. *Journal of Family Nursing*, 23(2), 175–200. 10.1177/1074840717696924 28795897 PMC5553294

[cit0040] Methley, A. M., Campbell, S., Chew-Graham, C., McNally, R., & Cheraghi-Sohi, S. (2014). PICO, PICOS and SPIDER: A comparison study of specificity and sensitivity in three search tools for qualitative systematic reviews. *BMC Health Services Research*, 14(1), 579. 10.1186/s12913-014-0579-0 25413154 PMC4310146

[cit0041] Mills-Koonce, W. R., Rehder, P. D., & McCurdy, A. L. (2018). The significance of parenting and parent-child relationships for sexual and gender minority adolescents. *Journal of Research on Adolescence*, 28(3), 637–649. 10.1111/jora.12404 30515946 PMC7087348

[cit0042] Mirković, V., & Jerković, I. (2021). Experiences of mothers of LGBTQ children in Serbia: What comes after coming out? *The Qualitative Report*. 10.46743/2160-3715/2021.4680

[cit0043] Murphy, T. F. (2018). Bioethics, children, and the environment. *Bioethics*, 32(1), 3–9. 10.1111/bioe.12386 28873213 PMC5763367

[cit0044] Newcomb, M. E., Feinstein, B. A., Matson, M., Macapagal, K., & Mustanski, B. (2018). I have No idea What’s going on out there:. *Parents’ Perspectives on Promoting Sexual Health in Lesbian, Gay, Bisexual, and Transgender Adolescents Sexuality Research and Social Policy*, 15(2), 111–122. 10.1007/s13178-018-0326-0 30245747 PMC6145819

[cit0045] Newhook, J. T., Winters, K., Msw, J. P., Feder, S., Holmes, C., Feder, S., Pickett, S., & Sinnott, M.-L. (2018). Teach your parents and providers well. *Canadian Family Physician Medecin de Famille Canadien*, 64(5), 332–335. 29760251 PMC5951646

[cit0046] Page, M. J., McKenzie, J. E., Bossuyt, P. M., Boutron, I., Hoffmann, T. C., Mulrow, C. D., Shamseer, L., Tetzlaff, J. M., Akl, E. A., Brennan, S. E., Chou, R., Glanville, J., Grimshaw, J. M., Hróbjartsson, A., Lalu, M. M., Li, T., Loder, E. W., Mayo-Wilson, E., McDonald, S. … Moher, D. (2021). The PRISMA 2020 statement: An updated guideline for reporting systematic reviews. *Systematic Reviews*, 10(1), 89. 10.1186/s13643-021-01626-4 33781348 PMC8008539

[cit0047] Pehrson, S., & Reicher, S. (2014). On the meaning, validity and importance of the distinction between personal and social identity: A social identity perspective on identity process theory. In G. M. Breakwell & R. Jaspal (Eds.), *Identity process theory: Identity, social action and social change* (pp. 97–117). Cambridge University Press. 10.1017/CBO9781139136983.009

[cit0048] Phillips, M. J., & Ancis, J. R. (2008). The process of identity development as the parent of a Lesbian or gay male. *Journal of LGBT Issues in Counseling*, 2(2), 126–158. 10.1080/15538600802125605

[cit0049] Reed, N. P., Josephsson, S., & Alsaker, S. (2020). A narrative study of mental health recovery: Exploring unique, open-ended and collective processes. *International Journal of Qualitative Studies on Health and Well-Being*, 15(1), 1747252. 10.1080/17482631.2020.1747252 32249712 PMC7170373

[cit0050] Rosati, F., Pistella, J., Nappa, M. R., & Baiocco, R. (2020). The coming-out process in family, social, and religious contexts among Young, middle, and older Italian LGBQ+ adults. *Frontiers in Psychology*, 11, 617217. 10.3389/fpsyg.2020.617217 33365008 PMC7750329

[cit0051] Rothman, E. F., Sullivan, M., Keyes, S., & Boehmer, U. (2012). Parents’ supportive reactions to sexual orientation disclosure associated with better health: Results from a population-based survey of LGB adults in Massachusetts. *Journal of Homosexuality*, 59(2), 186–200. 10.1080/00918369.2012.648878 22335417 PMC3313451

[cit0052] Rusi, J., Breakwell, G., & Deaux, K. (2016). *Identity process theory | social psychology*. Cambridge University Press. https://www.cambridge.org/in/academic/subjects/psychology/social-psychology/identity-process-theory-identity-social-action-and-social-change, https://www.cambridge.org/in/academic/subjects/psychology/social-psychology

[cit0053] Ryan, C., Russell, S. T., Huebner, D., Diaz, R., & Sanchez, J. (2010). Family acceptance in adolescence and the health of LGBT young adults: Family acceptance in adolescence and the health of LGBT young adults. *Journal of Child and Adolescent Psychiatric Nursing*, 23(4), 205–213. 10.1111/j.1744-6171.2010.00246.x 21073595

[cit0054] Sablonnière, R. D. L., & Usborne, E. (2014). Toward a social psychology of social change: Insights from identity process theory. In G. M. Breakwell & R. Jaspal (Eds.), *Identity process theory: Identity, social action and social change* (pp. 203–221). Cambridge University Press. 10.1017/CBO9781139136983.015

[cit0055] Saltzburg, S. (2004). Learning that an adolescent child is gay or lesbian: The parent experience. *Social Work*, 49(1), 109–118. 10.1093/sw/49.1.109 14964523

[cit0056] Savin-Williams, R. C. (1998). The disclosure to families of same-sex attractions by lesbian, gay, and bisexual youths. *Journal of Research on Adolescence*, 8(1), 49–68. 10.1207/s15327795jra0801_3

[cit0057] Sequeira, G. M., Ray, K. N., Miller, E., & Coulter, R. W. S. (2020). Transgender Youth’s disclosure of gender identity to providers outside of specialized gender centers. *Journal of Adolescent Health*, 66(6), 691–698. 10.1016/j.jadohealth.2019.12.010 PMC8496159 32089449

[cit0058] Spini, D., & Jopp, D. S. (2014). Old age and its challenges to identity. In *Identity process theory: Identity, social action and social change* (pp. 295–315). Cambridge University Press. 10.1017/CBO9781139136983.019

[cit0059] Spivey, L. A., Huebner, D. M., & Diamond, L. M. (2018). Parent responses to childhood gender nonconformity: Effects of parent and child characteristics. *Psychology of Sexual Orientation and Gender Diversity*, 5(3), 360–370. 10.1037/sgd0000279

[cit0060] Stotzer, R. L. (2011). Family cohesion among Hawai‘i’s *māhūwahine*. *Journal of GLBT Family Studies*, 7(5), 424–435. 10.1080/1550428X.2011.623935

[cit0061] Thornburgh, C., Kidd, K. M., Burnett, J. D., & Sequeira, G. M. (2020). Community-informed peer support for parents of gender-diverse youth. *Pediatrics*, 146(4), e20200571. 10.1542/peds.2020-0571 32973119 PMC7546091

[cit0062] Troub, R. M. (1983). General adjustment theory and institutional adjustment processes. *Journal of Economic Issues*, 17(2), 315–324. 10.1080/00213624.1983.11504114

[cit0063] Twigger-Ross, C. L., & Uzzell, D. L. (1996). PLACE and IDENTITY PROCESSES. *Journal of Environmental Psychology*, 16(3), 205–220. 10.1006/jevp.1996.0017

[cit0064] Umaña-Taylor, A. J. (2023). Promoting adolescent adjustment by intervening in ethnic-racial identity development: Opportunities for developmental prevention science and considerations for a global theory of change. *International Journal of Behavioral Development*, 47(4), 352–365. 10.1177/01650254231162614

[cit0065] Van Bergen, D. D., Wilson, B. D. M., Russell, S. T., Gordon, A. G., & Rothblum, E. D. (2021). Parental responses to coming out by Lesbian, gay, bisexual, queer, pansexual, or Two‐Spirited people across three age cohorts. *Journal of Marriage & Family*, 83(4), 1116–1133. 10.1111/jomf.12731 34413541 PMC8359215

[cit0066] Vázquez, I., Gato, J., Coimbra, S., Tasker, F., Barrientos, J., Miscioscia, M., Cerqueira-Santos, E., Malmquist, A., Seabra, D., Leal, D., Houghton, M., Poli, M., Gubello, A., Ramos, M. D. M., Guzmán-González, M., Urzúa, A., Ulloa, F., & Wurm, M. (2023). Psychological adjustment profiles of LGBTQ+ young adults residing with their parents during the COVID-19 pandemic: An international study. *International Journal of Environmental Research and Public Health*, 20(4), 3188. 10.3390/ijerph20043188 36833881 PMC9964666

[cit0067] Wagner, L. D., & Armstrong, E. (2020). Families in transition: The lived experience of parenting a transgender child. *Journal of Family Nursing*, 26(4), 337–345. 10.1177/1074840720945340 32744160

[cit0068] Watson, R. J., Rose, H. A., Doull, M., Adjei, J., & Saewyc, E. (2019). Worsening perceptions of family connectedness and parent support for lesbian, gay, and bisexual adolescents. *Journal of Child & Family Studies*, 28(11), 3121–3131. 10.1007/s10826-019-01489-3 31649475 PMC6812531

[cit0069] Zhou, Y., Furutani, M., Athurupana, R., & Nakatsuka, M. (2021). Relation between identity disclosure to family members and mental health in Japanese transgender people. *Acta Medica Okayama*, 75(5), 611–623. 10.18926/AMO/62774 34703044

